# The Effect of a Diet Containing Extruded Faba Bean Seeds on Growth Performance and Selected Microbial Activity Indices in the Large Intestine of Piglets

**DOI:** 10.3390/ani11061703

**Published:** 2021-06-07

**Authors:** Anna Tuśnio, Marcin Barszcz, Marcin Taciak, Ewa Święch, Agnieszka Wójtowicz, Jacek Skomiał

**Affiliations:** 1 Department of Animal Nutrition, The Kielanowski Institute of Animal Physiology and Nutrition, Polish Academy of Sciences, Instytucka 3, 05-110 Jabłonna, Poland; m.barszcz@ifzz.pl (M.B.); m.taciak@ifzz.pl (M.T.); e.swiech@ifzz.pl (E.Ś.); j.skomial@ifzz.pl (J.S.); 2 Department of Thermal Technology and Food Process Engineering, University of Life Sciences in Lublin, Głęboka 31, 20-612 Lublin, Poland; agnieszka.wojtowicz@up.lublin.pl

**Keywords:** legumes, faba bean, growth performance, microflora activity, extrusion cooking, piglets

## Abstract

**Simple Summary:**

Grain legumes are cultivated for food and feed purposes in all regions of the world. Legumes are the main source of protein for a large part of the world population where animal protein is hardly available. The potential of grain legumes is increasing primarily due to the genetic improvement of their agricultural and nutritional characteristics and expansion of organic farming. They are also fed to animals as a component of concentrates and on farms producing “organic” food. Therefore, studies on the composition, nutritional value and factors affecting quality of legume protein contribute to a more efficient utilization of seeds as feed and food ingredients. Faba bean is rich in both starch and protein and is an important alternative protein source in animal nutrition; however, its potential is not yet fully exploited, particularly in pig diets. The aim of the study was to evaluate the effect of diets containing various levels of extruded faba bean seeds on growth performance and selected microbial activity indices in the large intestine of pigs. Treatments with faba bean seeds did not negatively affect growth performance (except for the highest level of faba bean) and microbial activity in the large intestine, and can be applied in piglet diets.

**Abstract:**

The study investigated the effect of replacing soybean meal with extruded faba bean seeds on piglet growth performance and selected microbial activity indices in the large intestine. In total, 24, 35-day-old, healthy, castrated piglets of similar body weight were divided into four groups with six replicates. Animals in the control group (C) were fed with soybean meal without extruded faba bean seeds. In other experimental groups, pigs were fed diets with the addition of 20 (FB20), 25 (FB25) or 30% (FB30) extruded faba bean seeds instead of soybean meal and wheat starch. Growth performance, histology of the large intestine, short-chain fatty acids (SCFA) and ammonia concentration, as well as the activity of bacterial enzymes in digesta samples, were analyzed. The intake of the FB25 diet resulted in an increased feed:gain ratio in comparison to the FB30 group. Feeding the FB30 diet increased tunica muscularis thickness in the caecum as compared to other groups. Moreover, dietary inclusion of extruded faba bean seeds had no effect on SCFA and ammonia concentration. In addition, feeding diets with a different level of extruded faba bean seeds did not affect the activity of bacterial enzymes in the colon.

## 1. Introduction

Feeding-related costs are one of the most important factors affecting swine production. There is still a need for feedstuffs that would cover the nutritional requirements of animals and be economically profitable. Feed components should also have a good influence on animal health and welfare. Currently, soybean is widely used in diets for pigs because it has a good protein and amino acid profile, and it can be used with other plant feedstuffs rich in protein and energy [1]. It seems that faba bean (*Vicia faba* L., also known as broad bean or field bean), which can be grown in a cool, moist climate, and therefore in many places in Europe, could be such a valuable component of monogastric diets. Faba bean contains 27–34% protein in seed dry matter and a beneficial amino acid profile, but it also has protease inhibitors, lectins and tannins that reduce protein digestibility [2]. Moreover, the presence of such antinutritional factors in feed may adversely affect the intestinal mucosa, leading to the atrophy of intestinal villi [3]. Some researchers described negative effects of legumes on pig performance [4,5], but there have also been reports about positive effects on these parameters. Some authors indicated that a low concentration of several tannin sources improved the health status and performance of monogastric animals [6,7,8], and pigs are relatively insensitive to the detrimental effects associated with tannins. Growth improvement could be also obtained when certain technological processes (e.g., extrusion cooking, autoclaving) were applied to refine legume seeds, as they inactivated heat-labile, growth-depressing factors (protease inhibitors) [9,10]. Extrusion is also an effective process that modifies the structure of starch [11] and improves the nutritive value of feeds. However, tannins are thermo-stabile substances and vary in structure, composition and ability to bind and precipitate proteins [12]. Their chemical structure and concentration depend on temperature, light intensity, nutrient stress and exposure to herbivory [13]. In addition, the cultivar and color of faba bean flowers are also important, as they affect tannin content. White-flowered faba bean contains less than 1% tannins, while color-flowered plants have a higher tannin content.

The weaning period is a very important and critical moment in the piglet life [14]. Diet conversion from mother’s milk to solid mixtures often leads to post-weaning syndromes which change intestinal morphology and function [15]. Moreover, diet composition is a very important factor responsible for microbiota composition and function in the gut [16]. Legumes, being a source of fermentable carbohydrates (resistant starch and non-digestible oligosaccharides of the raffinose family), affect the intestinal microbiota by modulating its activity and composition, and lead to a higher microbial diversity of end-products of bacterial metabolism [17]. It is important for the microbial ecosystem, which is more stable when the diversity is higher [18]. Oligosaccharides influence, among others, the concentration of short-chain fatty acids (SCFA) [19] derived from bacterial fermentation. Approximately 95% of SCFA are readily absorbed during the transit of digesta through the colon, and butyrate is the major energy source for colonocytes, while acetic acid is the most abundant acid in the colon [20]. The large intestine is also the site of intensive protein turnover [21]. Dietary protein fermentation can lead to the production of branched-chain fatty acids and potentially toxic metabolites including ammonia, amines or phenols [22]. Ammonia can be absorbed into the blood and excreted in the urine or assimilated by bacteria. Its concentration in the colon depends on amino acid deamination and bacterial protein synthesis, and may possibly be involved in tumor promotion [23]. The activity of some bacterial enzymes is also important for colon health and animal welfare. Among them are mucinase, β-glucuronidase and β-glucosidase, which may increase the risk of carcinogenesis [24].

We hypothesized that partial replacement of soybean meal by extruded faba bean seeds (up to 30%) in the diets for piglets would be possible without negatively affecting the animals. Therefore, the aim of the present study was to investigate the effect of different levels of extruded faba bean seeds on growth performance and microbial activity in the large intestine of pigs.

## 2. Materials and Methods

The experiment was conducted in accordance with the guidelines of the Second Local Animal Experimentation Ethics Committee (resolution number WAW2/051/2018) at the Warsaw University of Life Sciences—SGGW, Warszawa, Poland.

Faba bean seeds cv. Bobas were purchased from a commercial supplier. Extrusion cooking of faba bean was performed using a TS-9/5 twin screw extruder–cooker (Z.M.Ch. Metalchem, Gliwice, Poland). Temperature zones were set at 115/135/140/140 °C and kept constant during the process. The extrusion-cooking process was performed at a screw speed of 80 rpm and the extrudates were shaped with a 5 mm open die. After extrusion cooking, the material was collected, cooled down to room temperature and stored in bags.

Twenty four castrated piglets (Danbred × Duroc), with body weight of 9.5 ± 0.25 kg at the beginning of the study, were used as experimental animals. The piglets were fed cereal-based diets without (control group) or with the addition of 20 (FB20), 25 (FB25) or 30% (FB30) extruded faba bean seeds instead of soybean meal and wheat starch (Table 1). Diets were formulated according to the Nutrient Requirements of Swine [25] and Nutrient Requirements and Nutritional Value of Feeds for Swine [26]. The animals were individually housed in pens for 21 days and had free access to feed and water throughout the study. Ambient temperature was controlled automatically and maintained at 25 ± 1 °C, and humidity was maintained at 60%. The light regime was a 12 h light/12 h dark cycle. Daily feed intake was recorded during the experimental period and body weight was measured once a week. At the end of the experiment, the animals were sacrificed, the large intestine was dissected and tissue and digesta samples were collected from the caecum and proximal, middle and distal part of the colon for histological measurements, SCFA and ammonia concentrations determination and selected bacterial enzyme activity (β-glucosidase and mucinase) analyses.

In the experimental diets, dry matter, crude protein (N × 6.25), crude ash, crude fiber, crude fat and acid detergent fiber expressed, excluding residual ash, were analyzed according to methods described in AOAC [27]. Neutral detergent fiber was analyzed with a heat-stable amylase and expressed, excluding residual ash, according to Mertens [28]. Condensed tannin content and trypsin inhibitor activity (TIA) (expressed as mg pure trypsin inhibited per gram of sample) in the experimental diets were determined colorimetrically according to the method of Jerumianis [29] with modifications of Adams and Novellie [30] and Kakade et al. [31], respectively.

Tissue samples for histological measurements were placed in buffered formalin (10%), then dehydrated and embedded in paraffin according to standard procedures. The samples were cut on a microtome—Microm 350 (Thermo Fisher Scientific, Walldorf, Germany) into 5 μm sections on two slides and subsequently stained with hematoxylin and eosin. An Olympus BX51 microscope (Olympus Corp., Tokyo, Japan) was used to measure crypt depth and tunica muscularis thickness. At least 20 measurements were obtained for each parameter.

Colonic digesta pH was measured using a digital pH meter—WTW pH/340 (Xylem Analytics Germany Sales GmbH & Co. KG, Weilheim, Germany). The pH was promptly adjusted to at least 8.2 with 1 M NaOH solution and the samples were centrifuged for 10 min at 1800 g. The supernatant was frozen and stored at −20 °C for further analyses. SCFA were analyzed according to the method described by Barszcz et al. [32] using a HP 5890 Series II gas chromatograph (Hewlett Packard, Waldbronn, Germany) with a flame-ionization detector. Isocaproic acid was used as an internal standard and separation was performed using a fused silica capillary column Supelco NUKOL (Supelco, Bellefonte, PA, USA) (internal diameter—30 m × 0.25 mm, film—0.25 mm).

Ammonia concentration in colonic digesta was measured spectrophotometrically using a Maxmat PL multidisciplinary diagnostic platform (Erba Diagnostics France SARL, Montpellier, France) according to the method of Taciak et al. [33] based on the reaction of ammonium ion with Nessler’s reagent. A standard curve was prepared with an ammonium chloride solution and absorbance was measured at 425 nm.

Bacterial enzyme activity (β-glucosidase and mucinase) was measured colorimetrically according to the methods described by Barszcz et al. [34].

Data were analyzed using one-way ANOVA and differences between means were determined by Tukey’s HSD test using STATGRAPHICS^®^ Centurion XVI ver.16.1.03 statistical software [35]. Normal data distribution was checked using Shapiro–Wilk test. The effects were considered statistically significant at *p* ≤ 0.05.

## 3. Results

Feed intake did not differ between the groups. Significant differences were observed only for the feed conversion ratio. Piglets fed the FB25 diet had a smaller feed:gain ratio than piglets from the FB30 group. In the FB25 group, a tendency of greater average daily body weight gain was observed compared to other groups (Table 2).

The large intestine morphology is presented in Table 3. Tunica muscularis in the caecum was significantly thicker in the FB30 group compared to the remaining experimental groups. Feeding pigs the FB30 diet also resulted in an increase in tunica muscularis thickness in the middle colon as compared to the control and FB20 groups. There were no differences in crypt depth in the caecum and colon segments between treatments.

Concentrations of SCFA are presented in Table 4. No differences in SCFA concentration were observed in any colon segment of pigs fed the experimental diets. Only iso-butyric acid concentration in the caecum tended to be lower in pigs fed diets with extruded faba bean seeds in relation to the control group, whereas acetic acid concentration in the middle colon tended to be reduced by the higher content of faba bean seeds in a diet.

Only a tendency towards lower ammonia concentration in the middle colon in the FB20 and FB30 groups was observed in comparison with the C and FB25 groups (Table 5). There were no statistically significant differences in the remaining sections of the large intestine.

There was no effect of dietary level of extruded faba bean on β-glucosidase activity in the large intestine of piglets (Table 6).

Dietary level of faba bean seeds had no significant effect on the mucinase activity in any parts of the large intestine (Table 7). Only a tendency was observed to a greater mucinase activity in the caecum of pigs fed the FB25 diet.

## 4. Discussion

Interest in using legumes in pig diets has increased in recent years [36,37], because they constitute an alternative plant protein source to soybean meal. The results show that extruded faba bean seeds are a valuable feed component in piglet nutrition. Weaned piglets fed the FB25 diet had a better feed:gain ratio than pigs fed the FB30 diet. Our results are consistent with the study of Brand [38], who did not observe a negative impact of faba bean on growth performance in piglets fed a diet containing 20% seeds. However, according to Emiola and Gous [39], the presence of 15% faba bean in a pig diet impaired growth performance and a 40% addition significantly reduced animal growth.

The structure of the intestinal mucosa is one of the most important elements indicating the health status of the gastrointestinal tract [40]. Ortiz et al. [41] conducted a study in chicks and found that histological lesions were associated with tannins derived from faba bean seeds, which could cause a loss of absorptive capacity. In the present study, we observed a greater thickness of tunica muscularis in the caecum and middle colon in the FB30 group as compared to the C, FB20, FB25 and C and FB20 groups, respectively. We also recorded a trend towards a greater thickness of tunica muscularis in the proximal colon in the FB25 group in comparison to the remaining groups. We suspected that these changes could be related to fiber level in a diet. Kissmeyer-Nilsen et al. [42] reported in a study on rats involving SCFA enemas that SCFA had a transmural trophic effect and protected the colonic mucosa from dysfunctions and atrophy. The current study shows that feeding piglets with experimental diets had no significant effect on SCFA concentration in the large intestine. Only a tendency to lower iso-butyrate concentration was observed in the caecum when pigs were fed diets with extruded faba bean seeds. Biagia et al. [7] suggested that tannins significantly reduced the concentration of ammonia, and iso-butyric and iso-valeric acids, which was associated with the reduction in bacterial proteolytic reactions in weaned pigs. In our study, acetic acid concentration also tended to be lower in the middle colon at higher levels of faba bean seeds in the diets. These results were in line with the in vitro analyses of Biagia et al. [7], who showed a negative correlation between dietary tannin content and caecal microbiota activity in pigs. The latter authors found that gas production and acetate, propionate and butyrate concentrations linearly decreased when tannin increased. These authors also suggested that total bacterial activity was reduced by tannins without inducing any favorable metabolic pathway. A similar observation was also made by Bravo et al. [43], who reported that tannic acid decreased acetic acid production and indicated its role in reducing the abundance of the acetic-acid-producing microflora.

Ammonia is the main end product of bacterial fermentation of nitrogenous compounds in the colon and can also be involved in de novo synthesis of bacterial protein [44]. Some authors indicated that ammonia could decrease SCFA utilization by intestinal epithelial cells, and they also found that excessive ammonia production could negatively impact the host [45,46,47]. In addition, intestinal epithelial cells can control ammonia concentration through conversion to citruline and glutamine, but also by slow release into the bloodstream [48,49]. Therefore, it is ambiguous how intense protein catabolism is necessary to achieve toxic ammonia concentration and how it varies between hosts [50]. Our results demonstrate that ammonia concentration in the piglet colon was not related to dietary level of extruded faba bean seeds, as no effect of the experimental diets was observed. This could be related to a rather low and balanced protein level in the diets.

A diet with faba bean seeds may have a favorable influence on the activity of the colonic microflora and affect the activity of bacterial enzymes. The activity of β-glucosidase contributes to the fermentation of resistant starch and higher SCFA production in the gut [51]. This enzyme may also lead to the fermentation of NSP-glucose of cellulosic and β-glucan origin [52]. In the present study, the activity of β-glucosidase and mucinase was not affected by the level of extruded faba bean seeds in piglet diets, and it was probably related to the low abundance of bacteria using glucosides or mucins as substrates. Furthermore, β-glucosidase is known for its both positive and negative health effects depending on the chemical structure of glycosides. On the one hand, the hydrolysis of plant glycosides may lead to the formation of products with greater antimutagenic, antioxidant, anticarcinogenic and immunomodulating potential than their glycosides [53]. On the other hand, β-glucosidase is involved in the formation of toxic aglycons from plant glycosides. These potentially harmful metabolites may not cause diseases in pigs, but may slow down growth performance or reduce feed utilization [54].

## 5. Conclusions

In conclusion, the results of the present study show that extruded faba bean seeds are a good source of protein in diets for weaned piglets and may partially replace soybean meal; however, their content should not exceed 25%. At 30% dietary level, extruded faba bean seeds may reduce feed efficiency and increase tunica muscularis thickness in the large intestine. Inclusion of 20–30% faba bean seeds in a diet has no negative impact on selected microbial activity indices.

## Figures and Tables

**Table 1:** Composition of experimental diets, g/kg.

Ingredients	Diet			
	C ^1^	FB20 ^2^	FB25 ^3^	FB30 ^4^
Wheat	240.0	240.0	240.0	240.0
Barley	285.0	285.0	285.0	285.0
Soybean meal	278.0	160.0	130.0	100.0
Extruded faba bean	-	200.0	250.0	300.0
Rapeseed oil	6.5	23.0	30.0	31.0
Limestone	10.0	10.0	10.0	10.0
Salt	3.5	3.5	3.5	3.5
Monocalcium phosphate	14.0	5.0	5.0	5.0
Mineral–vitamin premix ^5^	5.0	5.0	5.0	5.0
Wheat starch	145.0	48.0	21.0	-
L-Lys HCl (78%)	7.0	7.0	7.0	7.0
L-Met (98%)	2.0	2.5	2.5	2.5
L-Thr (98%)	3.0	3.0	3.0	3.0
L-Trp (98%)	1.0	1.0	1.0	1.0
Analyzed, g/kg				
Dry matter	896.0	897.2	898.3	899.8
Crude protein	201.2	196.9	203.7	196.9
Crude fat	24.8	37.2	42.3	43.6
Crude ash	51.2	48.1	49.4	46.1
Crude fiber	24.8	37.2	42.3	43.6
NDF ^6^	155.8	150.2	165.5	157.3
ADF ^7^	57.7	63.4	61.1	73.6
Tannins	0.142	0.210	0.244	0.267
TIA ^8^, mg/g	0.175	0.144	0.135	0.132
Calculated, g/kg				
EM, MJ/kg	13.3	13.4	13.4	13.4
Ca	8.1	7.5	7.5	7.4
P	6.7	6.6	6.7	6.8
SID Lys	13.4	13.4	13.3	13.3
SID Met	4.2	4.4	4.3	4.2
SID Thr	8.9	8.4	8.3	8.2
SID Trp	2.6	2.5	2.4	2.4

^1^ control diet; ^2^ diet with 20% extruded faba bean seeds; ^3^ diet with 25% extruded faba bean seeds; ^4^ diet with 30% extruded faba bean seeds; ^5^ provided per kilogram of diet: vit. A 20,000 IU; vit. D_3_ 2000 IU; vit. E 60 mg; vit. K_3_ 2 mg; vit. B_1_ 3 mg; vit. B_2_ 6 mg; vit. B_3_ 30 mg; pantothenic acid 1.22 mg; vit. B_6_ 4 mg; vit. B_12_ 40 µg; biotin 100 µg; vit. C 80 mg; folic acid 0.60 mg; Fe 240 mg; Mn 160 mg; Cu 40 mg; Zn 240 mg; I 3.20 mg; Co 1.60 mg; Se 0.80 mg; ^6^ neutral detergent fiber; ^7^ acid detergent fiber; ^8^ trypsin inhibitor activity.

**Table 2:** Growth performance of pigs fed diets with extruded faba bean seeds.

Item	Diet				SEM	*p*-Value
	C ^1^	FB20 ^2^	FB25 ^3^	FB30 ^4^		
Feed intake, kg	14.9	14.1	15.7	15.6	0.08	0.503
Live weight, kg	9.3	9.2	9.8	9.6	0.54	0.879
Average daily gain, kg	0.502	0.494	0.575	0.496	0.0230	0.060
Feed:gain, kg/kg	1.42 ^ab^	1.36 ^ab^	1.30 ^a^	1.49 ^b^	0.037	0.009

^1^ control group; ^2^ group with 20% extruded faba bean seeds; ^3^ group with 25% extruded faba bean seeds; ^4^ group with 30% extruded faba bean seeds; ^a^, ^b^—means in the rows with different letters differ significantly (*p* ≤ 0.05).

**Table 3:** Large intestine morphology of pigs fed diets with extruded faba bean seeds, μm.

Item	Diet				SEM	*p*-Value
	C ^1^	FB20 ^2^	FB25 ^3^	FB30 ^4^		
	Caecum					
Crypt depth	460	423	457	464	35.0	0.822
Tunica muscularis	531 ^a^	593 ^a^	587 ^a^	916 ^b^	70.3	0.005
	Proximal colon					
Crypt depth	440	454	472	454	26.5	0.867
Tunica muscularis	337	410	528	505	51.1	0.057
	Middle colon					
Crypt depth	485	450	516	517	30.0	0.361
Tunica muscularis	434 ^a^	446 ^a^	554 ^ab^	623 ^b^	38.0	0.006
	Distal colon					
Crypt depth	473	472	582	488	47.0	0.293
Tunica muscularis	424	512	534	488	37.3	0.256

^1^ control group; ^2^ group with 20% extruded faba bean seeds; ^3^ group with 25% extruded faba bean seeds; ^4^ group with 30% extruded faba bean seeds; ^a^, ^b^—means within rows with different letters differ significantly (*p* ≤ 0.05).

**Table 4:** SCFA concentration in the large intestine of pigs fed diets with extruded faba bean seeds, µmol/g digesta.

Item	Diet				SEM	*p*-Value
	C ^1^	FB20 ^2^	FB25 ^3^	FB30 ^4^		
	Caecum					
Acetic acid	36.2	35.7	36.9	40.6	1.66	0.191
Propionic acid	18.5	16.1	18.9	17.9	1.09	0.315
Butyric acid	9.96	8.69	9.67	7.77	0.893	0.345
Valeric acid	2.26	1.34	1.55	0.855	0.523	0.289
Iso-valeric acid	0.268	0.166	0.204	0.208	0.027	0.133
Iso-butyric acid	0.334	0.263	0.281	0.292	0.017	0.068
	Proximal colon					
Acetic acid	41.9	35.3	38.7	40.1	2.06	0.257
Propionic acid	21.7	15.2	19.0	18.4	1.53	0.117
Butyric acid	11.4	8.97	10.0	8.79	1.090	0.523
Valeric acid	2.90	1.56	1.74	1.32	0.445	0.256
Iso-valeric acid	2.90	1.56	1.74	1.32	0.445	0.256
Iso-butyric acid	0.330	0.237	0.308	0.309	0.0410	0.481
	Middle colon					
Acetic acid	32.6	36.9	39.6	33.4	1.96	0.071
Propionic acid	15.5	16.6	20.2	15.4	1.53	0.123
Butyric acid	8.32	10.6	11.5	9.45	1.109	0.232
Valeric acid	2.04	1.96	2.08	1.61	0.427	0.859
Iso-valeric acid	0.676	0.498	0.532	0.780	0.097	0.179
Iso-butyric acid	0.615	0.495	0.523	0.637	0.059	0.281
	Distal colon					
Acetic acid	32.9	30.4	32.2	28.8	1.59	0.267
Propionic acid	14.6	14.0	15.4	12.0	1.64	0.480
Butyric acid	8.12	8.89	9.06	8.12	1.423	0.942
Valeric acid	2.07	2.03	1.71	1.99	0.443	0.938
Iso-valeric acid	1.12	0.96	0.82	1.64	0.291	0.203
Iso-butyric acid	0.905	0.748	0.705	1.168	0.172	0.212

^1^ control group; ^2^ group with 20% extruded faba bean seeds; ^3^ group with 25% extruded faba bean seeds; ^4^ group with 30% extruded faba bean seeds.

**Table 5:** Ammonia concentration in the large intestine of pigs fed diets with extruded faba bean seeds, µmol/g digesta.

Item	Diet				SEM	*p*-Value
	C ^1^	FB20 ^2^	FB25 ^3^	FB30 ^4^		
Caecum	4.78	2.23	3.93	5.14	1.348	0.448
Proximal colon	7.19	4.62	8.12	7.85	1.756	0.508
Middle colon	12.56	7.20	14.65	8.29	2.103	0.071
Distal colon	12.99	12.25	6.54	7.90	2.520	0.235

^1^ control group; ^2^ group with 20% extruded faba bean seeds; ^3^ group with 25% extruded faba bean seeds; ^4^ group with 30% extruded faba bean seeds.

**Table 6:** β-glucosidase activity in the large intestine of pigs fed diets with extruded faba bean seeds, U/g digesta.

Item	Diet				SEM	*p*-Value
	C ^1^	FB20 ^2^	FB25 ^3^	FB30 ^4^		
Caecum	404	509	492	544	51.7	0.315
Proximal colon	483	481	570	606	60.9	0.395
Middle colon	583	687	773	679	74.8	0.509
Distal colon	556	743	682	619	69.1	0.286

^1^ control group; ^2^ group with 20% extruded faba bean seeds; ^3^ group with 25% extruded faba bean seeds; ^4^ group with 30% extruded faba bean seeds.

**Table 7:** Mucinase activity in the large intestine of pigs fed diets with extruded faba bean seeds, U/g digesta.

Item	Diet				SEM	*p*-Value
	C ^1^	FB20 ^2^	FB25 ^3^	FB30 ^4^		
Caecum	103	102	213	100	32.1	0.057
Proximal colon	135	199	223	124	38.6	0.206
Middle colon	130	156	145	111	15.0	0.236
Distal colon	154	150	221	175	31.0	0.365

^1^ control group; ^2^ group with 20% extruded faba bean seeds; ^3^ group with 25% extruded faba bean seeds; ^4^ group with 30% extruded faba bean seeds.

## Data Availability

The data presented in this study are available on request from the corresponding author.
